# Integrated Microfluidic Isolation of Aptamers Using Electrophoretic Oligonucleotide Manipulation

**DOI:** 10.1038/srep26139

**Published:** 2016-05-24

**Authors:** Jinho Kim, Timothy R. Olsen, Jing Zhu, John P. Hilton, Kyung-Ae Yang, Renjun Pei, Milan N. Stojanovic, Qiao Lin

**Affiliations:** 1 Department of Mechanical Engineering, Columbia University, New York, New York 10027, United States; 2 Division of Clinical Pharmacology and Experimental Therapeutics, Department of Medicine, Columbia University, Columbia University, New York, NY 10032, United States; 3 Division of Nanobiomedicine, Key Laboratory of Nano-Bio Interface, Suzhou Institute of Nano-Tech and Nano-Bionics, Chinese Academy of Sciences, Suzhou, Jiangsu 215123, China

## Abstract

We present a microfluidic approach to integrated isolation of DNA aptamers via systematic evolution of ligands by exponential enrichment (SELEX). The approach employs a microbead-based protocol for the processes of affinity selection and amplification of target-binding oligonucleotides, and an electrophoretic DNA manipulation scheme for the coupling of these processes, which are required to occur in different buffers. This achieves the full microfluidic integration of SELEX, thereby enabling highly efficient isolation of aptamers in drastically reduced times and with minimized consumption of biological material. The approach as such also offers broad target applicability by allowing selection of aptamers with respect to targets that are either surface-immobilized or solution-borne, potentially allowing aptamers to be developed as readily available affinity reagents for a wide range of targets. We demonstrate the utility of this approach on two different procedures, respectively for isolating aptamers against a surface-immobilized protein (immunoglobulin E) and a solution-phase small molecule (bisboronic acid in the presence of glucose). In both cases aptamer candidates were isolated in three rounds of SELEX within a total process time of approximately 10 hours.

Aptamers are synthetic oligonucleotide-based receptors that bind to biochemical targets^1^, and are isolated from libraries of randomized oligonucleotides through an iterative *in vitro* selection and amplification process termed systematic evolution of ligands by exponential enrichment (SELEX)^2^,^3^. Aptamers offer an attractive alternative to other receptors such as antibodies^4^, as they are synthetically available for a diverse range of targets (e.g., small molecules, peptides, amino acids, proteins, cells, viruses, and bacteria), are reproducible with minimal batch-to-batch variations, and are stable with excellent shelf life^2^,^5^,^6^,^7^. Aptamers can also be designed to bind to targets in specific functional domains^8^,^9^ or with predefined kinetic or equilibrium binding characteristics^10^,^11^,^12^,^13^. Additionally, aptamers are amenable to chemical conjugation with other molecules (e.g., diagnostic and therapeutic agents)^14^,^15^, and are potentially low in immunogenicity (when used *in vivo*) due to their small size^4^. Broad applications have been explored for aptamers in basic biological sciences, drug discovery, clinical diagnostics and therapeutics, and other fields^16^,^17^.

Conventional SELEX procedures involve repeated (10–20 rounds) execution of manual, tube-based processes such as mixing of oligonucleotides with targets, partitioning of binding oligonucleotides from non-binders, elution and amplification of binding oligonucleotides, and purification of the amplified product^2^,^3^. Completion of these processes typically require a month or longer of laborious work by highly trained personnel^1^,^18^. Robotic automation, while alleviating the labor-intensiveness of the manual procedures, is prohibitive and impractical for individual non-specialist laboratories^19^. Capillary electrophoresis-based methods can reduce the overall SELEX process time down to several days;^20^ however, they still require significant manual handling on the basis of a highly specialized set of skills, and are not well suited to very large (e.g., cell^7^) or small^21^ (e.g., small molecule) targets. As a result, aptamers are not yet routinely available for use in non-specialist laboratories.

There has been emerging effort to apply microfluidic technology to aptamer isolation. Microfluidic affinity selection has been performed, in conjunction with off-chip amplification, against targets retained using silica capillary walls^22^, microbeads^23^,^24^,^25^,^26^,^27^,^28^ or sol-gels^29^,^30^,^31^, or against cells in solution^32^,^33^, to increase selection stringency^23^,^24^,^25^,^26^,^27^,^32^,^33^, create more favorable biomolecular environments^29^,^30^,^31^, or allow simultaneous positive and negative selections^28^. Soh and coworkers, focusing solely on the affinity selection stage of SELEX, incubated oligonucleotides with target-functionalized magnetic beads, which were then purified in a continuous-flow fashion in a microchannel. This allowed highly efficient affinity selection of aptamer candidates, which were next amplified using off-chip PCR instrumentation. Similarly, Kim and coworkers performed affinity selection of oligonucleotides against targets such as proteins and small molecules that were immobilized in sol-gels. These oligonucleotides were next, again, amplified off-chip. Aiming to integrate the overall SELEX process, Lee and coworkers used pneumatically based flow control in microchips for isolation of aptamers targeting viruses^34^, cells^27^,^35^,^36^ and proteins^37^,^38^. While capable of performing individual affinity selection and PCR amplification processes on-chip, the microchips apparently required an off-chip procedure to retrieve single-stranded DNA from amplified products between successive rounds of SELEX, and thus did not allow full integration of SELEX. In addition, these devices were mostly limited to targets (e.g., proteins and cells) that are readily surface-immobilizable, and were in general not applicable to targets (e.g., small molecules) whose immobilization is difficult and against which isolation of aptamers is challenging^39^,^40^.

We present an approach to microfluidic SELEX that fully integrates all steps of the SELEX process on a single microchip. The approach is based on two engineering innovations: a fully bead-based protocol that applies to affinity selection and PCR amplification, and electrophoretic coupling of affinity selection and PCR amplification, two major phases of each SELEX round that are required to occur in different buffers. These innovations, which to our knowledge have not been demonstrated in existing microfluidic SELEX efforts, significantly simplify the manipulation of target molecules and oligonucleotides (e.g., retention, separation, and retrieval) as well as buffers (e.g., replenishment and replacement). More importantly, they critically enable the on-chip coupling of successive SELEX rounds and achieve the full microfluidic integration of the SELEX process, thereby enabling highly efficient isolation of aptamers in drastically reduced times and with minimized consumption of biological material. The bead-based SELEX protocol also affords broad target applicability by allowing selection of aptamers with respect to targets that are either surface-immobilized or solution-borne. As such, the approach is in principle applicable to virtually any form of target (e.g., small molecules, proteins, cells, viruses, and bacteria), potentially allowing aptamers to be developed as broadly applicable affinity reagents.

We demonstrate the utility of our microfluidic SELEX approach by isolating DNA aptamers in two procedures with (*i*) a target protein on microbeads and an oligonucleotide library in solution, and (*ii*) a small-molecule target in solution and an oligonucleotide library immobilized on microbeads. These protocols were successfully applied, respectively, to isolate oligonucleotides that bind to immunoglobulin E (IgE) protein on beads and oligonucleotides that are released from beads in the presence of bisboronic acid-glucose complexes (a modified Shinkai receptor-monosaccharide complex^41^). These results show the potential of our microfluidic SELEX approach for efficient, automated and rapid isolation of aptamers as readily available and broadly applicable affinity reagents in biomedical applications.

## Experimental Section

### Integrated Aptamer Isolation

**Figure 1:**
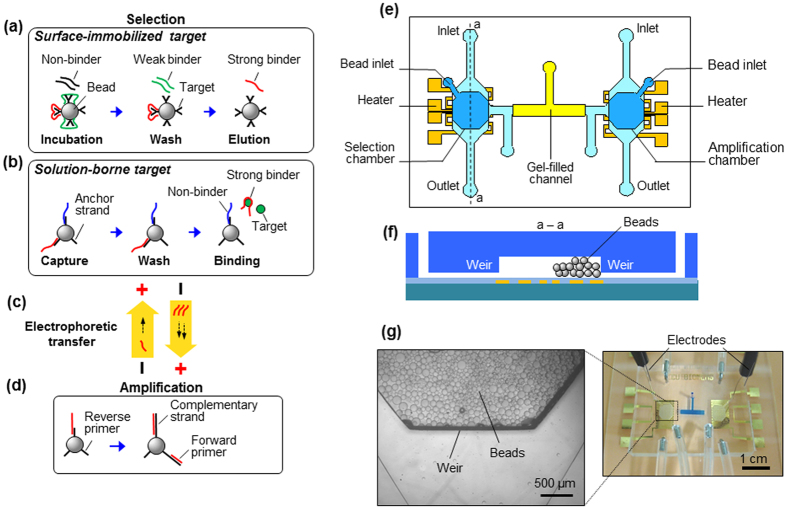
Integrated microfluidic SELEX. On microbeads, oligonucleotides are selected against a (**a**) surface-immobilized or (**b**) solution-phase target. Strong binders to the target are transferred away electrophoretically (**c**), and are subsequently captured and then amplified via PCR on microbeads (**d**), with the product transferred back electrophoretically to start a new round of affinity selection. This principle is implemented in a microchip consisting of the selection and amplification microchambers each integrated with a resistive heater and temperature sensor, and interconnected by a gel-filled microchannel: (**e**) top and (**f**) cross-sectional schematics. A micrograph of the microchip is shown in (**g**), with microbeads retained in the microchambers and agarose gel (dyed blue for visualization) filling the interconnection channel. Inset: Micrograph of the weir-like flow constriction region with retained beads.

Our approach to integrated aptamer isolation is based on a microchip, which consists of two microchambers (respectively referred to as the selection and amplification chambers, 5 μL each in volume) interconnected by a microchannel (length: 7 mm, width: 1 mm, height: 300 μm) (Fig. 1e). Each chamber is integrated with a weir-like flow constriction (weir height: 40 μm) to retain microbeads, and with a resistive micro heater and temperature sensor (Cr/Au: 5 nm/100 nm) for temperature control (Fig. 1f). The microchannel is filled with an agarose gel that allows electrophoresis of oligonucleotides while preventing bulk flow (Supplementary Information, Figure S3).

The entire SELEX process for aptamer isolation is integrated on the microchip, using a bead-based protocol for both affinity selection and amplification, via polymerase chain reaction (PCR), of target-binding oligonucleotides. Affinity selection can be performed against a target that is either surface-immobilized or solution-borne in the selection chamber. In the former case, a library of randomized single-stranded DNA (ssDNA) oligonucleotides in solution is incubated with a target (e.g., protein) that is attached to microbeads, allowing those strands of sufficient affinity to bind to the target. Following removal of weakly and non-binding strands with buffer wash, the strong binders are thermally released from the beads (Fig. 1a and Figure S1a). On the other hand, affinity selection for a target not amenable to surface-immobilization (e.g., a small molecule) occurs in solution phase. The solution-borne target is incubated with randomized oligonucleotides immobilized by hybridization to a short ssDNA strand that is attached to microbeads and serves as an anchor (Fig. 1b). Target-binding oligonucleotides are released from the surfaces if the oligomer-target affinity is greater than the oligomer-anchor affinity (Figure S1b). In both affinity selection schemes, target-binding oligonucleotides are released into the buffer and transferred, via electrophoretic transport through the gel-filled interconnecting channel, into the amplification chamber, and captured via hybridization by a reverse primer immobilized on microbeads therein^42^,^43^ (Fig. 1c).

The target-binding oligonucleotides are then amplified by PCR on microbeads^44^ (Fig. 1d and Supplementary Information Figure S2). The initial PCR cycle produces the complimentary strand to the target-binding oligonucleotides, resulting in double stranded DNA (dsDNA) attached to the beads. Subsequent cycles, additionally using a solution-borne forward primer, produce duplicate copies of the double stranded DNA (dsDNA). Thus, the final PCR product is in the form of bead-immobilized dsDNA where the target-binding oligonucleotides are hybridized to their complementary strands, which are in turn immobilized on bead surfaces. The dsDNA is split into single strands by chemical denaturation, releasing the amplified target-binding oligonucleotides into the solution while their complementary strands remain tethered to the bead surfaces. The amplified target binders are electrophoretically transported, again through the gel-filled interconnection channel, back into the selection chamber, where they are further affinity selected as described above. This procedure is repeated in multiple rounds to generate a pool of enriched target-binding oligonucleotides. Thus, the entire iterative SELEX process is integrated on a single chip and does not require any offline procedures, thanks to on-chip coupling of successive SELEX rounds as critically enabled by the bead-based PCR protocol. The enriched aptamer pools were cloned and sequenced to identify unique ssDNA sequences for each target.

### Microchip Fabrication and Testing

The microchip was fabricated of poly(dimethylsiloxane) (PDMS) for proof of principle, although other materials could readily be used to implement our approach. In the fabrication process, SU-8 photoresist was used to form a mold on a silicon substrate defining the microfluidic features. A prepolymer solution of PDMS was poured onto the mold, cured on a hotplate. The resulting PDMS sheet was released from the mold, and bonded onto a glass substrate that had been fabricated with Cr/Au heaters and temperature sensors. Finally, the interconnection channel between the chambers was filled with a molten 4% agarose gel and allowed to cure at room temperature (Fig. 1g).

In SELEX experiments, the target, ssDNA library, and PCR reagents were introduced into and removed from each microchamber using a syringe pump (NE-1000, New Era Pump Systems Inc.), at a flow rate of 10 μL/min unless otherwise noted. The pumps were manually controlled for demonstration of principle (although they could be readily programmed and automated, or replaced with established micropumps and valves^45^,^46^,^47^) and were not required for the coupling of successive rounds of SELEX, which was realized on-chip by the bead-based protocol as described above. The electric field (25 V/cm) for electrophoresis was generated in the interconnection channel by applying a voltage (from the power supply) to platinum (Pt)-wire electrodes inserted into the bead inlets. The temperature in each chamber was controlled within ±0.5 °C using the integrated heater and temperature sensors by a LabVIEW-based proportional-integral-derivative (PID) controller that was connected to a multimeter (34410A, Agilent Technologies) and a power supply (E3631A, Agilent Technologies) (Supplementary Information, Figure S4)^44^.

Affinity selection was performed at 25 °C. Microbeads functionalized with the target (for protein targets) or bearing the ssDNA library (for small-molecule targets) were injected into the selection chamber until they occupied approximately 50% of the chamber volume. Then the ssDNA library (1 μM in selection buffer, for protein targets) or target solution (1 μM in selection buffer, for small molecules) was infused into the chamber and incubated with the beads for 10 minutes. Additionally for protein targets, wash buffer was infused into the chamber (20 μL/min) to remove weakly binding ssDNA, followed by raising the chamber temperature to 57 °C to release protein binder into the solution. Next, reverse primer-functionalized beads were introduced into the amplification chamber to occupy approximately 50% of the chamber volume. An electric field was applied to electrophoretically transfer binding oligonucleotides to the amplification chamber. PCR reagents were then introduced into the chamber, followed by PCR thermal cycling (20 cycles) using on-chip temperature control. Meanwhile, the beads in the selection chamber were replaced with either fresh target functionalized beads (for protein targets) or beads with complimentary ssDNA anchors (for small-molecule targets). Following completion of PCR, 0.2 M NaOH was introduced into the amplification chamber to release the target-binding ssDNA, which was then transferred, using an an electric field, back to the selection chamber for affinity selection of the next SELEX round. The detailed on-chip SELEX procedure for protein and small molecule targets can be found in the Supporting Information (Tables S1 and S2). The process time, including the time to condition the microchip (e.g., introducing the gel) and to complete the SELEX process (from the introduction of beads to the output of the aptamer candidates), took approximately 10 hours. While the entire iterative SELEX process was performed on-chip, buffer washes were collected from the individual stages of each round, and oligonucleotides therein were amplified via off-chip PCR using a thermocycler (Eppendorf Mastercycler Gradient, Eppendorf) and analyzed via gel electrophoresis to characterize the SELEX process.

### Chemicals and Reagents

The randomized ssDNA library and primers were purchased from Integrated DNA Technologies. The DNA library used in aptamer selection for IgE protein were labeled with fluorescein (Excitation/Emission: 495 nm/520 nm) and contained a randomized region of 40 bases flanked by 24- and 23-base primer regions for the PCR amplification (5′-GCC TGT TGT GAG CCT CCT GTC GAA -40N -TTG AGC GTT TAT TCT TGT CTC CC-3′). The ssDNA library used in aptamer selection against bisboronic acid-glucose mixtures contained a randomized region of 30 bases flanked by 18- and 24-base primer regions (5′-GGA GGC TCTC GGG ACG AC -30N-GTC GTC CCG ATG CTG CAA TCG TAA-3′), while the corresponding anchor for bead immobilization was a biotinylated 19-mer ssDNA (3′-ATA TCC GAG AGC CCT GCT G-5′). NHS-activated agarose microbeads (diameter: 45–165 μm with a mean of 90 μm) and streptavidin-functionalized agarose microbeads (diameter: 50–80 μm) were obtained from GE Healthcare Life Sciences and Thermo Scientific, respectively. Human IgE and IgG proteins were purchased from Athens Research and Sigma-Aldrich, respectively. Chemicals for preparation of selection buffers (44.5 mM Tris base, 44.5 mM bisboronic acid, 50 mM NaCl, pH 8.5) and elution buffers (44.5 mM Tris base, 44.5 mM bisboronic acid, 50 mM NaCl, 0.2 M NaOH) for protein and small molecule were purchased from Sigma-Aldrich.

To prepare IgE-functionalized beads, NHS activated agarose beads (200 μL) were washed 3 times in a column with selection buffer. The beads were then incubated with 5.7 μM IgE (35 μL) at room temperature for 5 h on a shaker and washed 3 times with selection buffer. To block the NHS binding sites not occupied by IgE, the beads were incubated with 0.1M Tris-HCl buffer at room temperature for 1 h followed by washing 3 times with selection buffer. The IgE functionalized beads were stored in selection buffer in a refrigerator (4 °C). Microbeads for aptamer selection against bisboronic acid-glucose mixtures were prepared by incubating 500 pmol of biotinylated ssDNA anchor with 50 μL of streptavidin-functionalized agarose beads at room temperature for 30 minutes. Following the incubation, the beads were washed 3 times with selection buffer and incubated with 100 pmole of the ssDNA library for 30 minutes, which had previously been heated at 95 °C for 5 minutes and then held at room temperature for ~15 minutes. The beads were then washed with selection buffer and stored in a refrigerator. Similarly, beads for PCR amplification were prepared by incubating 100 pmole of biotinylated reverse primer with 50 μL of streptavidin-functionalized beads.

To prepare bisboronic acid-glucose molecule mixtures, 4 μL of 2.5 mM bisboronic acid was mixed with 10 μL of 1 M glucose in 186 μL of selection buffer. The final mixture of 50 μM bisboronic acid and 50 mM glucose was incubated for 20 minutes at room temperature before each experiment.

### Binding Measurements

A standard fluorescence-binding assay was used to measure the binding affinity of aptamer candidates to IgE. The fluorescently labeled enriched aptamer pool and individual sequences identified through sequencing were prepared at varying concentrations (0–100 nM) in selection buffer (total volume: 100 μL). IgE-functionalized beads in tubes (3 × 10^4^ per tube) were washed with selection buffer and incubated with the DNA strands at room temperature for 2 h. Following the incubation, the beads were washed with selection buffer three times to remove unbound oligonucleotides. The tubes containing beads were heated at 95 °C for 10 min. Oligonucleotides eluted from the beads were collected and quantified using a plate reader. The fluorescence intensity data were analyzed to estimate the dissociation constant (*K*_*D*_) by nonlinear regression using Origin (Origin Lab Corporation). To estimate binding affinity for the bisboronic acid-glucose mixture, microbeads functionalized with the enriched pool or with particular sequences from the pool were incubated with different concentrations of the mixture (0–12.5 μM) at room temperature for 30 minutes. Oligonucleotides released from the beads by binding to the target were collected and amplified. Gel images of the amplification product were obtained following gel electrophoresis, and the intensities of the gel bands were analyzed, using ImageJ software, to determine the amount of DNA released. The amount of DNA released was analyzed to estimate the dissociation constant using Origin.

## Results and Discussion

We first present results from characterization of the individual analytical procedures, and then those from the integrated aptamer isolation process as well as target-binding affinity measurements for the resulting aptamers. The IgE antibody was used^48^ as a representative surface-immobilized target, while bisboronic acid mixed with glucose (BA-glucose mixture) was used as a representative solution-borne target^39^.

### Affinity Selection

**Figure 2:**
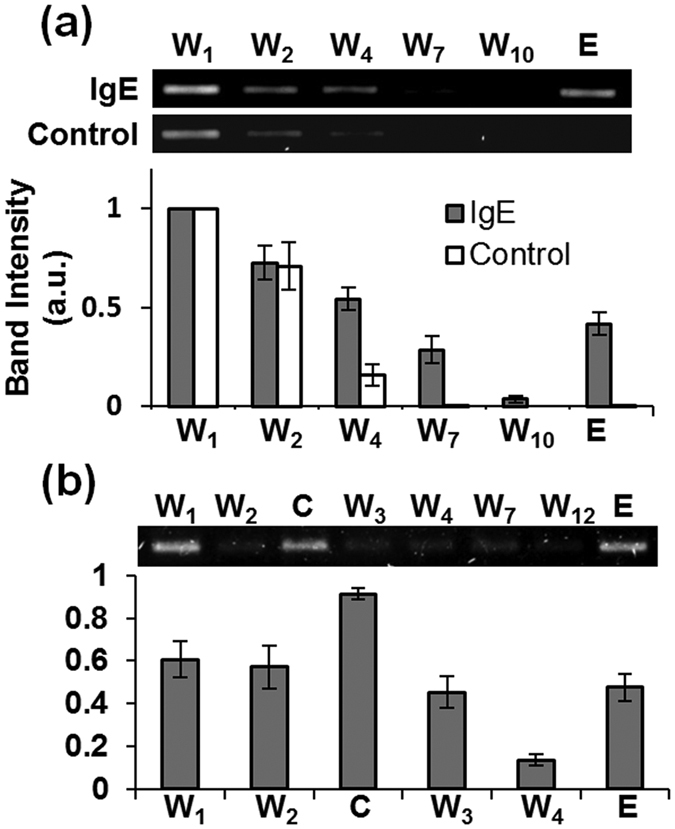
Microfluidic affinity selection of target-binding oligonucleotides. Bar graphs and representative gel images depict the relative band intensity in the gel image (inset) of eluate obtained during affinity selection for (**a**) IgE and (**b**) BA-glucose mixture. As control, bare beads were used for the affinity selection experiments for IgE. Bisboronic acid was used as a counter target for isolating aptamers targeting the BA-glucose mixture. Lane W: wash; Lane E: elution; Lane C: counter selection. All experiments were repeated three or more times (Supplementary Information, Figures S11, S12 and S13) with standard errors indicated with error bars.

We first characterized affinity selection of a randomized oligonucleotide library against targets. Eluates collected from the selection chamber during affinity selection were PCR amplified and analyzed using conventional gel electrophoresis (Fig. 2). The progress of affinity selection was investigated by comparing the fluorescence intensities of the bands in gel images, which were indicative of the amount of ssDNA in the eluates loaded in the corresponding gel lane.

For protein targets, the library was introduced into the selection chamber that contained protein-functionalized beads. The chamber was then washed with 10 buffer washes (~33 μL) to remove weakly binding ssDNA. The band intensity for the 10 buffer washes (W_1_–W_10_) in the gel image decreased with the wash number, indicating that weakly and non-binding oligonucleotides were successively removed from the microbeads and the chamber. On the other hand, the band intensity for the elution lane (E) increased to ~2.5 times that for the final wash (W_10_) as oligonucleotides that strongly bound to the bead-immobilized IgE were released. These oligonucleotides specifically bound to IgE rather than nonspecifically adsorbed to the chamber or bead surfaces, as no visible band was seen for the elution lane in the control experiment performed using bare beads (Fig. 2a).

For affinity selection against the BA-glucose mixture, the library was introduced into the selection chamber, which had been preloaded with capture beads. Oligonucleotides not captured by the bead-immobilized anchor were removed from the chamber during two initial buffer washes and collected (W_1_ and W_2_). Then a counter target (bisbornonic acid) was introduced and the eluate was collected. The band with relatively high fluorescence intensity for the eluate obtained during counter selection (C) indicated that strands that bound only to the bisboronic acid (used as a counter target) were eliminated. Oligonucleotides that still remained in solution were removed with continuous buffer washes (W_3_–W_12_), followed by the release and elution of target-binding oligonucleotides upon introduction of the target molecule, as indicated by the strong intensity for the elution band (E), which was ~6 times greater than that for the final wash (W_12_) (Fig. 2b).

### Electrophoretic Oligonucleotide Transfer

**Figure 3:**
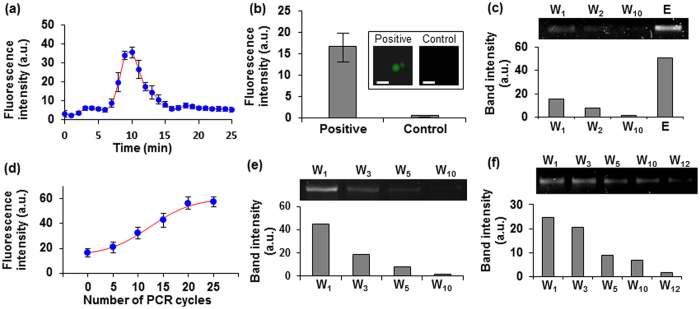
Characterization of electrophoretic transfer and bead-based PCR amplification of target-binding oligonucleotides. (**a**) Fluorescence intensity measurements at the center of the gel-filled interconnection channel as fluorescently labeled IgE-binding oligonucleotides migrated from the selection chamber to the amplification chamber. (**b**) Fluorescence measurements of IgE-binding strands that were electrophoretically transferred to and captured by bead-immobilized reverse primers in the amplification chamber. Scale bars: 100 μm. (**c**) Gel electropherogram of oligonucleotides targeting the BA-glucose mixture eluted from the chip, following their electrophoretic transfer to, capture by, and subsequent thermal release from bead-immobilized reverse primers in the amplification chamber. (**d**) Fluorescence intensity of IgE-binding oligonucleotides amplified on beads following different numbers of PCR cycles. Affinity selection of PCR-amplified oligonucleotides against (**e**) IgE and (**f**) the BA-glucose mixture. Lane W: wash and Lane E: elution.

We characterized electrophoretic transfer of target-binding oligonucleotides from the selection chamber to the amplification chamber, and their subsequent capture onto microbeads. This process will be used to enable the on-chip coupling of affinity selection and amplification of target-binding oligonucleotides in any given SELEX round. Fluorescently labelled oligonucleotides, obtained from affinity selection against IgE in the selection chamber, were electrophoretically driven through the gel-filled interconnection channel into the amplification chamber. The fluorescent intensity, and hence the amount, of oligonucleotides passing by the channel’s midpoint increased steadily from the time *t* = 7 min following the application of the electric field. The amount of electrophoresed oligomers maximized at *t* = 10 min, and then gradually decreased but remained visible until *t* = 15 min (Fig. 3a). In addition, the oligonucleotides were captured by reverse primers immobilized on microbeads in the amplification chamber, as indicated by the strong fluorescence intensity of the beads compared to that of bare beads used as control (Fig. 3b).

Similarly, oligonucleotides selected using the BA-glucose mixture were electrophsoretically transferred and captured onto microbeads placed in the amplification chamber, as a strong band was seen in the first elution lane (E) in the gel image obtained using the eluates following the electrophoretic transfer and capture processes (Fig. 3c). In the experiments, no significant damage to the gel was observed even after the electric field was applied for as long as ~180 minutes, suggesting that electrophoretic transfer of oligonucleotides could be performed repeatedly in the chip as required by the multi-round SELEX process (Supplementary Information, Figure S5).

### Bead-Based PCR and On-Chip Coupling of Successive SELEX Rounds

We characterized bead-based PCR and its use to enable on-chip coupling of PCR amplification in one SELEX round and affinity selection in the immediately following SELEX round. Oligonucleotides captured by the bead-immobilized reverse primers were amplified via bead-based PCR using fluorescently labeled forward primers in the amplification chamber (Supplementary Information, Figure S2). The fluorescence intensity of the bead-bound dsDNA PCR product exhibited a sigmoidal dependence on the number of PCR thermal cycles, as typically observed in PCR on solid surfaces^49^ (Fig. 3d). During the initial ~10 PCR cycles, the bead fluorescence intensity increased exponentially. Between ~10 and ~20 PCR cycles, the bead fluorescence intensity increased much more slowly, indicating a decreased rate of PCR product generation likely due to a decreasing amount of bead-immobilized reverse primers available for the reaction^50^. Beyond ~20 cycles, the increase in fluorescence intensity became insignificant as the majority of reverse primers had already been converted into dsDNA PCR product. Thus, the maximum amount of oligonucleotides, which would be used for further affinity selection, could be produced using approximately 25 PCR cycles in our chips. Although a greater number of PCR cycles was used than usual, overamplification of non-specific binders was not considered significant^51^ because the amount of oligonucleotides amplified on beads was typically much smaller than conventional solution-based PCR^52^. We also verified that the amplification chamber could be repeatedly used for amplifying target-binding oligonucleotides during the iterative aptamer isolation processes (Supplementary Information, Figure S6).

To couple PCR amplification in one SELEX round with affinity selection in the next round, the oligonucleotides amplified in the amplification chamber were transferred electrophoretically back to the selection chamber, where they were further affinity selected. The bright bands in the gel images for the eluate collected during the first buffer wash (W_1_) following the application of the electric field indicated that this new round of affinity selection was successful for IgE (Fig. 3e) and for the BA-glucose mixture (Fig. 3f). For the affinity selection towards IgE, the gradual decrease in the band intensity with the buffer wash number shows the removal of oligonucleotides that less strongly bound to the target compared with those that remained on beads. For the affinity selection against BA-glucose mixture, the band intensity decreased with the mixture washes, showing that the target competitively bound to and released ssDNA from bead-immobilized anchors.

### Multi-Round Microfluidic SELEX

**Figure 4:**
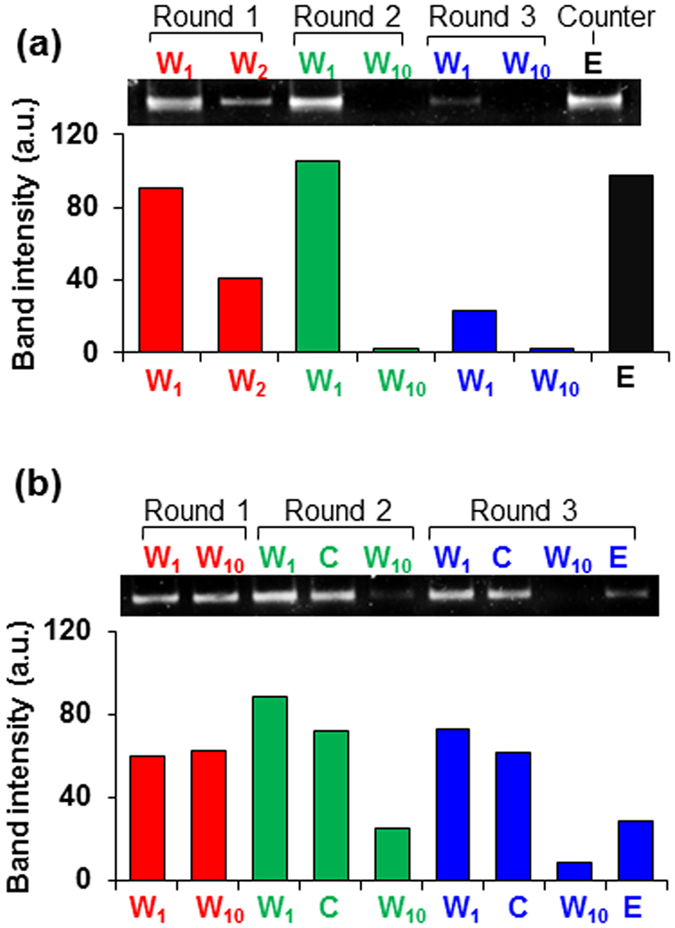
Gel electropherograms showing progress in the isolation of target-binding aptamer candidates during multi-round microfluidic SELEX. Bar graphs depict the band intensity in the gel images from eluents collected during the SELEX process for (**a**) IgE and (**b**) BA-glucose mixture. Lane W: wash; Lane C: counter selection; Lane E: elution.

Having characterized the individual procedures, we tested integrated multi-round SELEX for isolation of DNA aptamers. To minimize potential issues associated with electrolysis-induced pH changes of buffers during the electrophoretic transfer process^53^, fresh selection buffer was infused (1 μL/min) continuously into the chip’s anode port (Supplementary Information, Figure S7). Using a microchip, the isolation of protein-binding aptamer candidates was demonstrated with three rounds of affinity selection and amplification against IgE, followed by counter selection against human immunoglobulin G (IgG), to which the aptamer candidates were sought not to bind (Figure S8a). With an increasing number of buffer washes, the band intensities decreased from the initial wash (W_1_) to the final wash (W_10_) by approximately 55%, 98%, and 91% in SELEX rounds 1, 2, and 3, respectively, indicating the removal of weakly and non-binding oligonucleotides. The weaker band intensity for the initial wash (W_1_) in round 3 compared with that in rounds 1 and 2 could be attributed to the improvement in the affinities of oligonucleotides to IgE. Following counter selection against IgG, a pool of enriched IgE-binding oligonucleotides was obtained, as indicated by a distinct band (E) (Fig. 4a).

Three rounds of microfluidic SELEX were also performed using the BA-glucose mixture, with counter selection against bisboronic acid alone included in rounds 2 and 3. In round 1, oligonucleotides that were not captured by the bead-immobilized anchor were removed from the selection chamber (W_1_–W_10_). Discernible bands for the counter target (C) in rounds 2 and 3 indicated that strands that bound to bisboronic acid alone were removed. Oligonucleotides remaining in solution in the chamber were removed with further buffer washes, as indicated by the band intensity for the final wash (W_10_) from rounds 2 and 3 decreasing by approximately 71% and 88% relative to the initial wash (W_1_) of each round. The band intensity for the elution lane (E) was approximately three times that for the final wash (W_10_) in round 3, confirming that oligonucleotides that could bind to BA-glucose mixture were successfully isolated (Fig. 4b and Supplementary Information, Figure S8).

### Affinity of Aptamers

**Figure 5:**
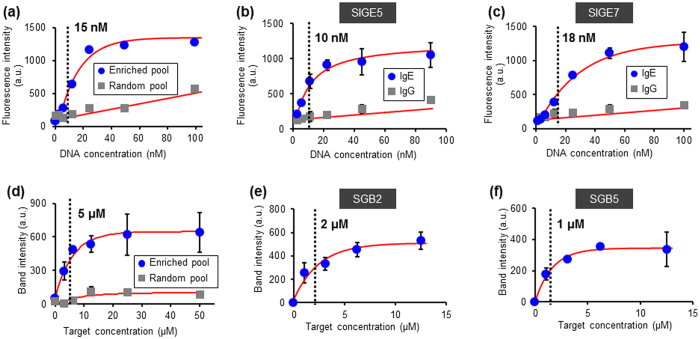
Target-binding affinity of aptamer candidates resulting from microfluidic SELEX. Fluorescence intensity was measured of thermally released oligonucleotides that bound to bead-immobilized IgE: (**a**) the enriched aptamer candidate pool and randomized oligonucleotide library, and the sequences (**b**) SIGE5 and (**c**) SIGE7 selected from the enriched pool. Fluorescence intensity was obtained for gel electrophoresis bands of oligonucleotides released from bead-immobilized ssDNA anchor due to binding to BA-glucose mixture: (**d**) the enriched aptamer candidate pool and randomized library, and the sequences (**e**) SBG2 and (**f**) SBG5 selected from the enriched pool.

We measured the affinity of the enriched aptamer candidate pools that resulted from microfluidic SELEX, as well as individual sequences selected from the pools (Supplementary Information, Figure S10). For IgE-targeting aptamers, we used a fluorescence binding assay in which target immobilized on beads were exposed to varying concentrations of fluorescently labeled oligonucleotides, and the amount of oligonucleotides initially bound to the targets were thermally released and quantitatively estimated^25^. For the enriched pool of IgE-binding aptamer candidates, the fluorescence intensity, indicative of the amount of IgE-oligonucleotide complexes, increased rapidly and reached saturation at low oligonucleotide concentrations (below ~30 nM). The equilibrium dissociation constant (*K*_*D*_) of the enriched aptamer candidate pool to the immobilized IgE was estimated to be 15 ± 1.5 nM. The fluorescence intensity from control experiments using the randomized oligonucleotide library versus IgE increased very slowly with the oligonucleotide concentration (Fig. 5a), indicating negligible target-binding affinity of the library. Moreover, fluorescence measurements of the enriched pool versus IgG (data not shown) showed negligible binding, verifying that the oligonucleotides in the pool were specific to IgE. The oligonucleotide sequences SIGE5 and SIGE7, which have a hairpin loop secondary structure, (Supplementary Information, Figure S9), from the pool showed strong binding to IgE with *K*_*D*_ = 10 ± 2.1 nM and *K*_*D*_ = ~18 ± 3.6 nM, respectively. These affinities are comparable to anti-IgE aptamers (*K*_*D*_ = ~10–35 nM^48^) isolated using conventional SELEX methods. The sequences were also determined to be specific to IgE by showing negligible affinity to the counter target IgG (Fig. 5b,c). The improved binding interactions between targets and oligonucleotides within microscale volumes, as suggested by previous studies involving single-step aptamer selection in small volumes^25^,^54^, could be responsible for aptamers to be successfully isolated in the small number (three) of rounds.

We also estimated the affinity of aptamer candidates isolated against the BA-glucose mixture as the target. The enriched aptamer candidate pool or sequences selected from the pool were initially captured by the bead-immobilized ssDNA anchor, and then released by exposure to different concentrations of the BA-glucose mixture^55^. The fluorescence intensity from measurements using the enriched aptamer candidate pool (Fig. 5d) as well as the selected sequences SBG2 (Fig. 5e and Figure S9c) and SBG5 (Fig. 5f and Supplementary Information, Figure S9) exhibited a rapid increase at target concentrations below ~5 μM followed by a slow increase at higher target concentrations. In comparison, the fluorescence intensity from control experiments using randomized oligonucleotides was substantially lower at all target concentrations, confirming the affinity of the aptamer candidates toward the BA-glucose mixture. The half-maximum concentration, i.e., the target concentration at which the fluorescence intensity reaches 50% of the saturation value (as achieved at high target concentrations)^56^, was approximately 5 ± 1.7 μM. Similarly, the half-maximum concentration for SBG2 and SBG5 against the BA-glucose mixture was estimated to be 2 ± 0.7 μM and 1 ± 0.5 μM, respectively, also suggesting strong interactions with the target. Further studies would be required to confirm that the oligonucleotides obtained from the microfluidic SELEX processes can function as solution-phase aptamers^57^ for glucose.

## Conclusions

We described a microfluidic approach to integrated isolation of aptamers against biological targets. The approach employs a microbead-based protocol for the processes of affinity selection and amplification of target-binding oligonucleotides, and an electrophoretic oligonucleotide manipulation scheme for effective two-way coupling of these processes, which are required to occur in different buffers. The entire iterative SELEX process is hence integrated on a single chip and does not require any offline procedures, thereby enabling highly efficient isolation of aptamers in drastically reduced times and with minimized consumption of biological material. In addition, the approach as such affords broad target applicability by allowing selection of aptamers with respect to targets that are either surface-immobilized or solution-borne, potentially allowing aptamers to be developed as readily available affinity reagents for a wide range of targets.

We demonstrated the utility of this microfluidic SELEX approach on two different procedures, respectively for isolating aptamers against immunoglobulin E as a representative surface-immobilized protein, and bisboronic acid-glucose mixture as a representative solution-borne target. For both targets, the SELEX process was completed in three rounds within a total process time of approximately 10 hours. The resulting aptamer candidates exhibited strong target-binding affinity, with an equilibrium dissociation constant of 18 nM or better toward immunoglobulin E, and a half-maximum concentration of 2 μM or better toward the bisboronic acid-glucose mixture.

The microfluidic SELEX experiments were performed on a PDMS-based microchip. While the adequacy of PDMS as the proof-of-concept material was indicated by the consistent and repeatable results from repeated experiments as well as the successful isolation of aptamer candidates, other microchip materials could readily be used to offer desired properties for specific intended applications. Additionally, the microchip could be further instrumented and programmed to allow fully automated operation. Thus, we envision that the microfluidic SELEX approach can enable broader use of aptamers by potentially allowing low-cost, rapid and automated discovery of oligonucleotide-based receptors for a diverse range of targets.

## Additional Information

**How to cite this article**: Kim, J. *et al.* Integrated Microfluidic Isolation of Aptamers Using Electrophoretic Oligonucleotide Manipulation. *Sci. Rep.* **6**, 26139; doi: 10.1038/srep26139 (2016).

## Supplementary Material

Supplementary Information

## References

[b1] Ellington A. D. & Szostak J. W. *In vitro* selection of RNA molecules that bind specific ligands. Nature 346, 818–822 (1990). 1697402 10.1038/346818a0

[b2] Jayasena S. D. Aptamers: an emerging class of molecules that rival antibodies in diagnostics. Clin. Chem. 45, 1628–1650 (1999). 10471678

[b3] Stoltenburg R., Reinemann C. & Strehlitz B. SELEX—a (r) evolutionary method to generate high-affinity nucleic acid ligands. Biomol. Eng. 24, 381–403 (2007). 17627883 10.1016/j.bioeng.2007.06.001

[b4] Gilboa E., McNamara J. & Pastor F. Use of oligonucleotide aptamer ligands to modulate the function of immune receptors. Clin. Cancer. Res. 19, 1054–1062 (2013). 23460536 10.1158/1078-0432.CCR-12-2067

[b5] Bunka D. H. & Stockley P. G. Aptamers come of age–at last. Nat. Rev. Microbiol. 4, 588–596 (2006). 16845429 10.1038/nrmicro1458

[b6] Gopinath S. C. B. Methods developed for SELEX. Anal. Bioanal. Chem. 387, 171–182 (2007). 17072603 10.1007/s00216-006-0826-2

[b7] Shamah S. M., Healy J. M. & Cload S. T. Complex Target SELEX. Acc. Chem. Res. 41, 130–138 (2008). 18193823 10.1021/ar700142z

[b8] Stojanovic M. N., De Prada P. & Landry D. W. Aptamer-based folding fluorescent sensor for cocaine. J. Am. Chem. Soc. 123, 4928–4931 (2001). 11457319 10.1021/ja0038171

[b9] Hamula C. L., Guthrie J. W., Zhang H., Li X.-F. & Le X. C. Selection and analytical applications of aptamers. TrAC, Trends Anal. Chem. 25, 681–691 (2006). 10.1016/j.trac.2011.08.006 PMC7112775 32287535

[b10] Berezovski M., Musheev M., Drabovich A. & Krylov S. N. Non-SELEX selection of aptamers. J. Am. Chem. Soc. 128, 1410–1411 (2006). 16448086 10.1021/ja056943j

[b11] Drabovich A., Berezovski M. & Krylov S. N. Selection of smart aptamers by equilibrium capillary electrophoresis of equilibrium mixtures (ECEEM). J. Am. Chem. Soc. 127, 11224–11225 (2005). 16089434 10.1021/ja0530016

[b12] Nguyen T., Pei R., Stojanovic M. & Lin Q. An aptamer-based microfluidic device for thermally controlled affinity extraction. Microfluid. Nanofluid. 6, 479–487 (2009).

[b13] Nguyen T., Pei R., Landry D. W., Stojanovic M. N. & Lin Q. Label-free microfluidic characterization of temperature-dependent biomolecular interactions. Biomicrofluidics 5, 034118 (2011). 10.1063/1.3620417 PMC3170392 21915242

[b14] Chu T. C., Twu K. Y., Ellington A. D. & Levy M. Aptamer mediated siRNA delivery. Nucleic Acids Res. 34, e73–e73 (2006). 16740739 10.1093/nar/gkl388 PMC1474074

[b15] McNamara J. O. *et al.* Multivalent 4-1BB binding aptamers costimulate CD8+ T cells and inhibit tumor growth in mice. J. Clin. Invest. 118, 376–386 (2008). 18060045 10.1172/JCI33365 PMC2104483

[b16] Green L. S., Bell C. & Janjic N. Aptamers as reagents for high-throughput screening. Bio Techniques 30, 1094–1100 (2001). 10.2144/01305dd02 11355345

[b17] Nimjee S. M., Rusconi C. P. & Sullenger B. A. Aptamers: an emerging class of therapeutics. Annu. Rev. Med. 56, 555–583 (2005). 15660527 10.1146/annurev.med.56.062904.144915

[b18] Tuerk C. & Gold L. Systematic evolution of ligands by exponential enrichment: RNA ligands to bacteriophage T4 DNA polymerase. Science 249, 505–510 (1990). 2200121 10.1126/science.2200121

[b19] Cox J. C., Rudolph P. & Ellington A. D. Automated RNA selection. Biotechnol. Prog. 14, 845–850 (1998). 9841645 10.1021/bp980097h

[b20] Mendonsa S. D. & Bowser M. T. *In vitro* evolution of functional DNA using capillary electrophoresis. J. Am. Chem. Soc. 126, 20–21 (2004). 14709039 10.1021/ja037832s

[b21] Yang J. & Bowser M. T. Capillary electrophoresis–SELEX selection of catalytic DNA aptamers for a small-molecule porphyrin target. Anal. Chem. 85, 1525–1530 (2013). 23234289 10.1021/ac302721j PMC3568998

[b22] Hybarger G., Bynum J., Williams R. F., Valdes J. J. & Chambers J. P. A microfluidic SELEX prototype. Anal. Bioanal. Chem. 384, 191–198 (2006). 16315013 10.1007/s00216-005-0089-3

[b23] Lou X. *et al.* Micromagnetic selection of aptamers in microfluidic channels. Proc. Natl. Acad. Sci. 106, 2989–2994 (2009). 19202068 10.1073/pnas.0813135106 PMC2637280

[b24] Qian J., Lou X., Zhang Y., Xiao Y. & Soh H. T. Generation of highly specific aptamers via micromagnetic selection. Anal. Chem. 81, 5490–5495 (2009). 19480397 10.1021/ac900759k PMC2704263

[b25] Cho M. *et al.* Quantitative selection of DNA aptamers through microfluidic selection and high-throughput sequencing. Proc. Natl. Acad. Sci. 107, 15373–15378 (2010). 20705898 10.1073/pnas.1009331107 PMC2932614

[b26] Weng C.-H., Lien K.-Y., Yang S.-Y. & Lee G.-B. A suction-type, pneumatic microfluidic device for liquid transport and mixing. Microfluid. Nanofluid. 10, 301–310 (2011).

[b27] Weng C.-H. *et al.* An automatic microfluidic system for rapid screening of cancer stem-like cell-specific aptamers. Microfluid. Nanofluid. 14, 753–765, 10.1007/s10404-012-1095-3 (2013).

[b28] Wang Q. *et al.* Screening of DNA Aptamers against Myoglobin Using a Positive and Negative Selection Units Integrated Microfluidic Chip and Its Biosensing Application. Anal. Chem. 86, 6572–6579 (2014). 24914856 10.1021/ac501088q

[b29] Park S.-M. *et al.* Selection and elution of aptamers using nanoporous sol-gel arrays with integrated microheaters. Lab Chip 9, 1206–1212 (2009). 19370238 10.1039/b814993c

[b30] Ahn J.-Y. *et al.* Sol–Gel Derived Nanoporous Compositions for Entrapping Small Molecules and Their Outlook toward Aptamer Screening. Anal. Chem. 84, 2647–2653 (2012). 22283623 10.1021/ac202559w

[b31] Lee S. *et al.* A cross-contamination-free SELEX platform for a multi-target selection strategy. Bio Chip Journal 7, 38–45 (2013).

[b32] Birch C. M., Hou H. W., Han J. & Niles J. C. Identification of malaria parasite-infected red blood cell surface aptamers by inertial microfluidic SELEX (I-SELEX). Sci. Rep. 5, 10.1038/srep11347 (2015). PMC4486934 26126714

[b33] Stoll H. *et al.* Microfluidic chip system for the selection and enrichment of cell binding aptamers. Biomicrofluidics 9, 034111 (2015). 26180568 10.1063/1.4922544 PMC4474950

[b34] Lai H.-C., Wang C.-H., Liou T.-M. & Lee G.-B. Influenza A virus-specific aptamers screened by using an integrated microfluidic system. Lab Chip 14, 2002–2013 (2014). 24820138 10.1039/c4lc00187g

[b35] Hung L.-Y., Wang C.-H., Hsu K.-F., Chou C.-Y. & Lee G.-B. An on-chip Cell-SELEX process for automatic selection of high-affinity aptamers specific to different histologically classified ovarian cancer cells. Lab Chip 14, 4017–4028 (2014). 25144781 10.1039/c4lc00587b

[b36] Hung L.-Y. *et al.* Screening of aptamers specific to colorectal cancer cells and stem cells by utilizing On-chip Cell-SELEX. Sci. Rep. 5, 10.1038/srep10326 (2015). PMC4650677 25999049

[b37] Lin H.-I. *et al.* Selection of aptamers specific for glycated hemoglobin and total hemoglobin using on-chip SELEX. Lab Chip 15, 486–494, 10.1039/C4LC01124D (2014). 25408102

[b38] Huang C.-J., Lin H.-I., Shiesh S.-C. & Lee G.-B. An integrated microfluidic system for rapid screening of alpha-fetoprotein-specific aptamers. Biosensors Bioelectron. 35, 50–55 (2012). 10.1016/j.bios.2012.02.024 22410487

[b39] Yang K.-A. *et al.* Recognition and sensing of low-epitope targets via ternary complexes with oligonucleotides and synthetic receptors. Nature Chem. 6, 1003–1008, 10.1038/nchem.2058 (2014). 25343606 PMC4339820

[b40] McKeague M. & DeRosa M. C. Challenges and opportunities for small molecule aptamer development. Journal of nucleic acids 2012, 10.1155/2012/748913 (2012). PMC3488411 23150810

[b41] James T. D., Sandanayake K. & Shinkai S. A Glucose‐Selective Molecular Fluorescence Sensor. Angewandte Chemie International Edition in English 33, 2207–2209 (1994).

[b42] Kim J. *et al.* Nucleic Acid Isolation and Enrichment on a Microchip. Sensors and Actuators A: Physical 195, 183–190 (2012). 24729660 10.1016/j.sna.2012.07.022 PMC3979544

[b43] Erickson D., Liu X., Krull U. & Li D. Electrokinetically controlled DNA hybridization microfluidic chip enabling rapid target analysis. Anal. Chem. 76, 7269–7277 (2004). 15595869 10.1021/ac049396d

[b44] Hilton J. P. *et al.* Bead-based polymerase chain reaction on a microchip. Microfluid. Nanofluid. 13, 749–760 (2012). 10.1007/s10404-012-0993-8 PMC7929480 33664642

[b45] Unger M. A., Chou H.-P., Thorsen T., Scherer A. & Quake S. R. Monolithic microfabricated valves and pumps by multilayer soft lithography. Science 288, 113–116 (2000). 10753110 10.1126/science.288.5463.113

[b46] Ni J., Huang F., Wang B., Li B. & Lin Q. A planar PDMS micropump using in-contact minimized-leakage check valves. Journal of Micromechanics and Microengineering 20, 095033 (2010). 24511208 10.1088/0960-1317/20/9/095033 PMC3915938

[b47] Sun H. *et al.* A bead-based microfluidic approach to integrated single-cell gene expression analysis by quantitative RT-PCR. RSC advances 5, 4886–4893 (2015). 25883782 10.1039/C4RA13356K PMC4394375

[b48] Wiegand T. W. *et al.* High-affinity oligonucleotide ligands to human IgE inhibit binding to Fc epsilon receptor I. The Journal of Immunology 157, 221–230 (1996). 8683119

[b49] Adessi C. *et al.* Solid phase DNA amplification: characterisation of primer attachment and amplification mechanisms. Nucleic Acids Res. 28, e87–e87 (2000). 11024189 10.1093/nar/28.20.e87 PMC110803

[b50] Higuchi R., Fockler C., Dollinger G. & Watson R. Kinetic PCR analysis: real-time monitoring of DNA amplification reactions. Biotechnology 11, 1026–1030 (1993). 7764001 10.1038/nbt0993-1026

[b51] Marimuthu C., Tang T.-H., Tominaga J., Tan S.-C. & Gopinath S. C. Single-stranded DNA (ssDNA) production in DNA aptamer generation. Analyst 137, 1307–1315 (2012). 22314701 10.1039/c2an15905h

[b52] Zhang Y. & Ozdemir P. Microfluidic DNA amplification—a review. Anal. Chim. Acta 638, 115–125 (2009). 19327449 10.1016/j.aca.2009.02.038

[b53] Zhang J., Lang H. P., Yoshikawa G. & Gerber C. Optimization of DNA hybridization efficiency by ph-driven nanomechanical bending. Langmuir 28, 6494–6501 (2012). 22439593 10.1021/la205066h

[b54] Schütze T. *et al.* Probing the SELEX process with next-generation sequencing. PLos One 6, e29604 (2011). 22242135 10.1371/journal.pone.0029604 PMC3248438

[b55] Jing M. & Bowser M. T. Methods for measuring aptamer-protein equilibria: a review. Anal. Chim. Acta 686, 9–18 (2011). 21237304 10.1016/j.aca.2010.10.032 PMC3026478

[b56] Tahiri-Alaoui A. *et al.* High affinity nucleic acid aptamers for streptavidin incorporated into bi-specific capture ligands. Nucleic Acids Res. 30, e45–e45 (2002). 12000850 10.1093/nar/30.10.e45 PMC115299

[b57] Cho E. J., Lee J.-W. & Ellington A. D. Applications of aptamers as sensors. Annu. Rev. Anal. Chem. 2, 241–264 (2009). 10.1146/annurev.anchem.1.031207.112851 20636061

