# Supplementary material for: Integrated Microfluidic Isolation of Aptamers Using Electrophoretic Oligonucleotide Manipulation
Source: Sci Rep. 2016 May 24;6:26139. doi: 10.1038/srep26139 (PMC4877600; doi:10.1038/srep26139)
Supplement: Supplementary Information [file srep26139-s1.pdf]

Supporting Information

Integrated Microfluidic Isolation of Aptamers Using Electrophoretic Oligonucleotide Manipulation

Jinho Kim,[†,§] Timothy R. Olsen,[†,§] Jing Zhu,[†] John P. Hilton,[†] Kyung-Ae Yang,[‡] Renjun Pei,[∥] Milan N. Stojanovic,[‡] and Qiao Lin*,[†]

[†]Department of Mechanical Engineering, Columbia University, New York, New York 10027, United States

[‡]Division of Clinical Pharmacology and Experimental Therapeutics, Department of Medicine, Columbia University, Columbia University, New York, NY 10032, United States

[∥]Division of Nanobiomedicine, Key Laboratory of Nano-Bio Interface, Suzhou Institute of Nano-Tech and Nano-Bionics, Chinese Academy of Sciences, Suzhou, Jiangsu 215123, China

Contents

1. DNA library and material S-2
2. Preparation of microbeads for selection and PCR amplification S-3
3. Preparation of the bisboronic acid sensor-glucose molecule mixture S-3
4. Device fabrication S-4
5. Microchip design S-4
6. Experimental setup S-4
7. Affinity selection of aptamer S-5
8. Electrophoretic transfer of oligonucleotides from the selection to amplification chamber S-6
9. Capture of oligonucleotides on reverse primer-coated beads S-7
10. Bead-based PCR of oligonucleotides S-7
11. Electrophoretic transfer of oligonucleotides amplified on beads from the amplification to selection chamber S-8
12. Multi-cycle SELEX S-8
13. Control of pH during electrophoretic transfer during multi-cycle SELEX S-8
14. Binding affinity measurement S-9
15. Supporting Figures S-10
16. Supplementary References S-19

1. DNA library and material

The randomized ssDNA library and primer strands were purchased from Integrated DNA Technologies. Each strand of the DNA library used in SELEX experiments for IgE protein was labeled with fluorescein (Excitation/Emission: 495 nm/520 nm) and contained a random region of 40 bases flanked by 24- and 23-base primer regions for the PCR amplification (5' - GCC TGT TGT GAG CCT CCT GTC GAA - 40N - TTG AGC GTT TAT TCT TGT CTC CC - 3'). The DNA library used for SELEX of the bisboronic acid sensor-glucose molecule mixture contains a random region of 30 bases flanked by 18- and 24-base primer regions (5' - GGA GGC TCTC GGG ACG AC -30N- GTC GTC CCG ATG CTG CAA TCG TAA - 3'), while the probe strand (3' - ATA TCC GAG AGC CCT GCT G - 5') used was biotinylated. NHS-activated microbeads (diameter: 45-165 μm, mean diameter: 90 μm) and streptavidin beads (diameter: 50-80 μm) were obtained from GE Healthcare Life Sciences and Thermo Scientific, respectively. Human IgE and IgG proteins were purchased from Athens Research and Sigma-Aldrich, respectively. Chemicals to prepare selection buffers for protein and small molecule (44.5 mM Tris base, 44.5 mM bisboronic acid sensor, 50 mM NaCl, pH 8.5) were purchased from Sigma-Aldrich. A power supply (E3631A, Agilent Technologies) and a multimeter (34410A, Agilent Technologies) controlled by LabVIEW software (National Instruments Corp.) running on a computer manipulated the temperature in the chip for PCR amplification on-chip. A conventional thermocycler (Eppendorf Mastercycler Gradient, Eppendorf) was used to amplify DNA strands for the gel electropherogram and large scale PCR.

2. Preparation of microbeads for selection and PCR amplification

To prepare IgE functionalized beads, NHS activated beads (200 μL) were washed 3 times in a column with selection buffer. The beads were then incubated with 5.7 μM IgE (35 μL) at room temperature for 5 hr on a shaker and were washed 3 times with selection buffer. To block the NHS binding sites not occupied by IgE, the beads were incubated with 0.1M Tris-HCl buffer at room temperature for 1 hr followed by buffer wash. The IgE functionalized beads were stored in selection buffer in a refrigerator (4°C). Microbeads for aptamer selection against the bisboronic acid sensor-glucose molecule mixture were prepared by incubating 500 pmol of capture strands with 50 μL of streptavidin beads at room temperature for 30 minutes. Following the incubation, the beads were washed 3 times with selection buffer and incubated with 100 pmole of DNA library for 30 minutes which was heated at 95°C for 5 minutes and cooled to room temperature. The beads were then washed with selection buffer and stored in a refrigerator. Similarly, beads for PCR amplification were prepared by incubating 100 pmole of biotinylated reverse primer with 50 μL of streptavidin beads.

3. Preparation of the bisboronic acid sensor-glucose molecule mixture

To prepare the bisboronic acid sensor-glucose molecule mixture, 4 μL of 2.5 mM bisboronic acid sensor was mixed with 10 μL of 1M glucose in 186 μL of selection buffer. The final mixture of 50 μM bisboronic acid sensor and 50 mM glucose was incubated for 20 minutes at room temperature before experiment. Fresh solution of the bisboronic acid sensor-glucose molecule mixture was prepared prior to each experiment.

4. Device fabrication

To prepare microfluidic layers of the devices, SU-8 molds were first prepared using photolithography. Then, poly(dimethylsiloxane) (PDMS) prepolymer was poured onto the molds, baked on a hotplate, and released. The microfluidic layers were bonded on glass substrates integrated with Cr/Au resistive heaters following oxygen plasma treatment. Molten 4% agarose gel was filled in the microchannel connecting two chambers in the device.

5. Microchip design

Integrated isolation of aptamers is achieved on a microchip. The chip consists of two microchambers (volume: 5 μL each) respectively for affinity selection and amplification of target-binding oligonucleotides. The chambers are each integrated with micro resistive heaters and temperature sensors (Cr/Au: 5/100 nm) for closed-loop temperature control, and a weir-shaped microstructure for retaining microbeads (weir height: 40 μm) and cells (weir height: ~8 μm). While reagent handling within each chamber is via pressure-driven fluid flow through the inlets and outlets, binding oligonucleotides are transferred between the chambers via electrophoresis through a section of agarose gel (length: 7 mm, height: 300 μm).

6. Experimental setup

Buffers and sample solutions were introduced into the device via reagent inlets using a syringe infusion pump (NE-1000, New Era Pump Systems Inc.). Approximately 50% of chamber volume was filled with beads through bead inlets. Eluents from the chambers

were collected at the outlets for analysis. The temperature in a chamber was controlled using the integrated heater and temperature sensors by a computer with PID controller connected to a multimeter (34410A, Agilent Technologies) and a power supply (E3631A, Agilent Technologies). An electric field for electrophoretic DNA transfer was generated on the chip using platinum (Pt)-wire electrodes inserted into the bead inlets under voltage from a power supply. All experiments were repeated three or more times and representative gel images are shown.

7. Affinity selection of aptamer

Affinity selection against IgE was characterized via incubation of the oligomer library (100 μM, 100 μL) with the microbead-attached protein, followed by removal of weakly binding oligonucleotides with buffer wash (10 μL/min) and then by the elution of strongly IgE-binding oligonucleotides from the beads by heating at 57°C for 5 min. Affinity selection against the bisboronic acid sensor-glucose mixture was performed by collecting (10 μL/min) oligonucleotides released from microbead-immobilized probe strands upon incubation with the target molecule. Eluates (~33 μL) obtained during buffer washes and elution of weakly- and strongly cell-binding oligonucleotides, respectively, were collected in separate tubes at the outlet of the selection chamber, amplified via PCR off-chip, and analyzed by gel electrophoresis for characterization of the affinity selection process. Control experiments for affinity selection of IgE were performed to verify that oligonucleotides selected were those that bound to the intended targets rather than those resulting from nonspecific adsorption to microbeads or chamber surfaces. For the IgE protein, control affinity selection experiments were performed with

the selection chamber containing NHS-activated agarose microbeads not functionalized with IgE. Such control experiments for investigation of nonspecific adsorption were not performed for the bisboronic acid sensor-glucose mixture because oligonucleotides released from the microbeads were necessarily a result of the binding interaction between the bisboronic acid sensor-glucose mixture and the oligonucleotides on the beads. Instead, the selection chamber was washed rigorously with buffer to ensure removal of oligonucleotides that might have non-specifically adsorbed onto the chamber surface during the experiments. To examine whether the selected oligonucleotides specifically bound to the bisboronic acid sensor-glucose mixture, however, a counter selection step using bisboronic acid sensor as a counter target was included between buffer washes.

8. Electrophoretic transfer of oligonucleotides from the selection to amplification chamber

Electrophoretic transfer of oligonucleotides was achieved by applying an electric field (25 V/cm) between the selection and amplification chambers. The migration of strands in the microchip was monitored using fluorescently labeled oligonucleotides via fluorescence microscopy. Fluorescence images were taken using a microscope (IX-81, Olympus) at the center of the gel-filled channel with a 1-minute time interval as the fluorescently labeled strands migrated from the selection to amplification chamber through the gel-filled channel. Then the fluorescence intensities of each image were measured over the duration of DNA transfer.

9. Capture of oligonucleotides on reverse primer-coated beads

To monitor the capture of oligonucleotides selected against IgE protein, the electrophoretically transferred oligonucleotides were incubated with microbeads, which were then washed with buffer to remove non-captured strands and measured for the fluorescence intensity. On the other hand, capture of oligonucleotides selected against the bisboronic acid sensor-glucose mixture was assessed via gel electrophoresis using the PCR product of the released strands that were captured by bead-immobilized reverse primers.

10. Bead-based PCR of oligonucleotides

Following capture of the electrophoretically transferred oligonucleotides on beads, the amplification chamber was washed with PBS buffer and filled with PCR reagents (GoTaq® Flexi DNA polymerase, Promega), including fluorescently labeled forward primers, and thermocycled to induce PCR (denaturation: 92°C for 15 s, annealing: 59°C for 30 s, elongation 72°C for 45 s). In the characterization experiments, oligonucleotides were affinity-selected in the selection chamber, electrophoretically transferred into and captured onto reverse primer-functionalized microbeads in the amplification chamber, and amplified on-chip via a varying number (5, 10, 15, 20 or 25) of PCR cycles. The microbeads were then washed to remove non-incorporated fluorescent forward primers and examined off-chip under a fluorescent microscope. Thus, the intensity of fluorescence signal measured from the beads presented the amount of affinity-selected aptamer candidates amplified on the bead surfaces.

11. Electrophoretic transfer of oligonucleotides amplified on beads from the amplification to selection chamber

The amplified dsDNA on microbeads were separated into single strands by incubating the beads with buffer containing NaOH (Elution buffer). The strands released into the buffer were then electrophoretically transferred into the selection chamber, where they underwent affinity selection against IgE or the bisboronic acid sensor-glucose mixture. Gel images of the eluates collected during buffer wash steps from the chip were used to assess the success of the electrophoretic transfer of the PCR product.

12. Multi-cycle SELEX

Multiple SELEX cycles were achieved by repeating the microchip-based aptamer isolation processes including affinity binding, electrophoretic transfer, and amplification. The eluates in the intermediate affinity selection processes, along with the eluate from the chip at the end of the SELEX process, were collected, amplified via off-chip PCR, and analyzed by gel electrophoresis. IgG protein was used as a counter target for aptamer isolation against IgE protein.

13. Control of pH during electrophoretic transfer during multi-cycle SELEX

To prevent potential issues associated with the change of the pH level at the anode during electrophoretic transfer of IgE-binding oligonucleotides in the microchip, the fresh selection buffer was continuously infused (flow rate: 1 μL/min) using a syringe pump. On the other hand, the bisboronic acid sensor-glucose mixture was continuously introduced

into the selection chamber (flow rate: 1 μL/min) during affinity-selection and electrophoretic transfer processes.

14. Binding affinity measurement

A standard fluorescence-binding assay was used to measure binding affinity of DNA strands to IgE. Fluorescently labeled strands with various concentrations (e.g., 0-100 nM) were prepared in selection buffer (total volume: 100 μL). IgE-functionalized beads in tubes (3×10^4/tube) were washed with selection buffer and incubated with the DNA strands at room temperature for 2 h. Following the incubation, the beads were washed with selection buffer three times to remove unbound strands. The tubes containing beads were heated at 95ºC for 10 min using a thermocycler. Eluted strands from the beads were collected and their amounts were measured using a plate reader. The fluorescence intensity data were analyzed to estimate the dissociation constant (K_D) by nonlinear curve fitting using the software Origin (Origin Lab Corporation). For the estimation of binding affinity for the bisboronic acid sensor-glucose mixture, microbeads functionalized with ssDNA strands were incubated with different concentrations of the bisboronic acid sensor-glucose mixture (0-12.5 μM) at room temperature for 30 minutes. The strands released from the beads by binding to the target were collected and amplified using a conventional thermocycler. Gel images of the amplified strands were obtained following gel electrophoresis from which the band intensity of each band was measured to determine the amount of DNA released from the beads incubated with different target concentrations. The band intensities were plotted for curve fitting to estimate half-maximum values of the signal.

15. Supporting Figures

ImageJ software (NIH) was used to measure the intensity of the band in gel images, and the fluorescence intensity of microbeads and the gel-filled microchannel in microscopic images.

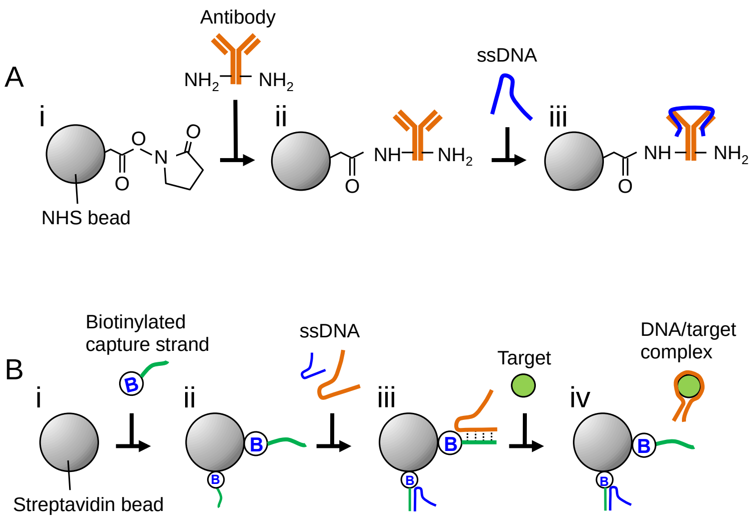

Fig. S1. (*A*) Functionalization of beads with an antibody target: (i) Incubation of NHS beads with an antibody target. (ii) Amide binding conjugates the antibody target onto the bead's surface. (iii) ssDNA strands that bind to the antibody can be captured to the bead immobilized target. (*B*) Small molecule functionalization and selection strategy. (i) Washing of streptavidin functionalized microbeads with buffer. (ii) Biotinylated capture strands bind to bead's surface. (iii) Hybridization of ssDNA strands to the bead-bound capture strands. (iv) Upon introduction of a target, target-binding strands will be released from the capture strands while strands that do not bind to the target will remain bound to the beads.

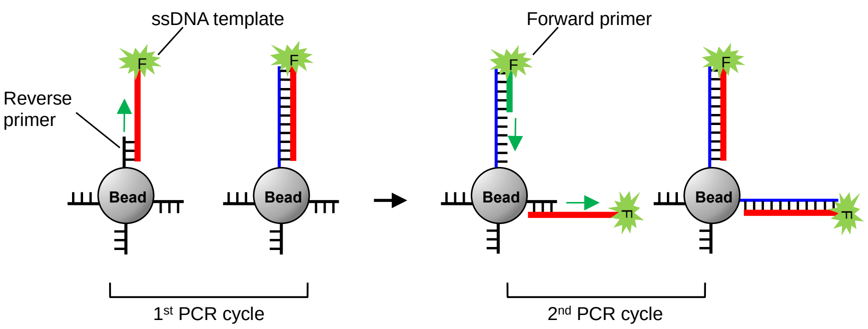

Fig. S2. A schematic showing bead-based PCR. During the 1st PCR cycle, an ssDNA strand hybridizes to a bead-bound reverse primer in the amplification chamber. DNA polymerase extends the reverse primer to produce a strand complementary to the template strand. During the 2nd PCR cycle, template strands will hybridize to another reverse primer and will produce a complementary strand. Forward primers will hybridize to the complementary strand produced during the 1st PCR cycle and will be extended to make an exact copy of the template strand. Using a fluorescent tag on the forward primers, the progress of bead-based PCR cycle can be monitored by measuring the fluorescence intensity of the beads.

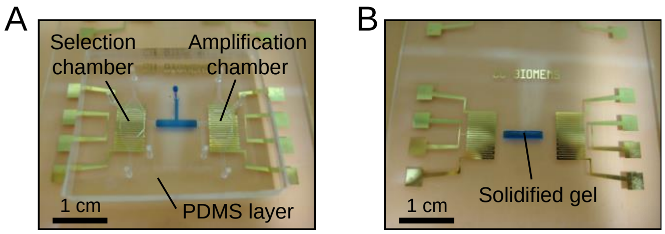

Fig. S3. Gel filled microchannel connecting the two chambers. (*A*) Molten 4% agarose gel was injected into the channel through the gel inlet. (*B*) The gel solidifies in the channel within 10 minutes. Blue ink was added to the gel for visualization and the PDMS layer was reversibly bonded onto the heater substrate to show the gel solidified in the channel.

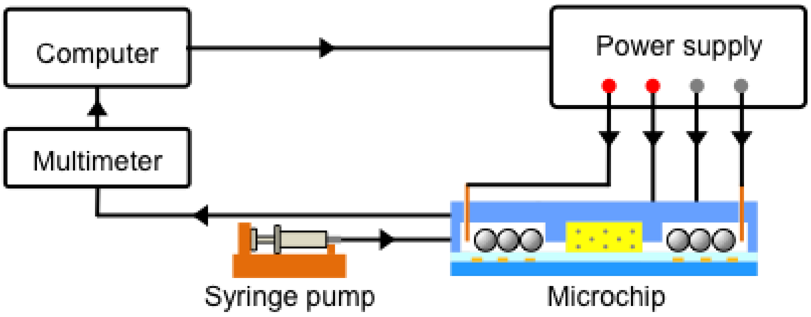

Fig. S4. Experimental setup of aptamer isolation using the chips. Buffers and samples were introduced using a syringe pump, while temperature was control using a computer-controlled PID setup.

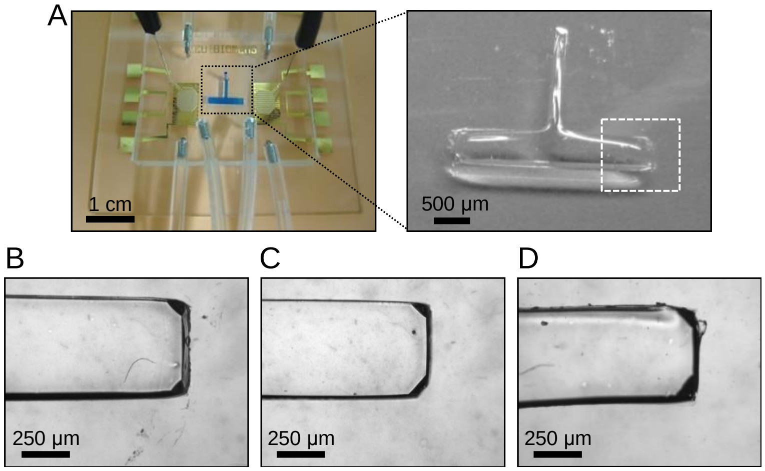

Fig. S5. Electric field effect on gel (*A*) Chip with PDMS layer reversibly bonded on the heater substrate. Gel dyed with blue ink for visualization. Gel used in actual experiments did not have dye added. Zoomed image shows a solidified gel placed on a glass substrate after removing the PDMS layer. (*B*) A micrograph image of a solidified gel that has not been exposed to an electric field. Region used for visual inspection indicated in the zoomed image of (*A*). Micrograph images of the same region of the gel following (*C*) 30 minutes and (*D*) 180 minutes of exposure to a 25 V/cm electric field. The gels maintained their shape following exposure to an electric field.

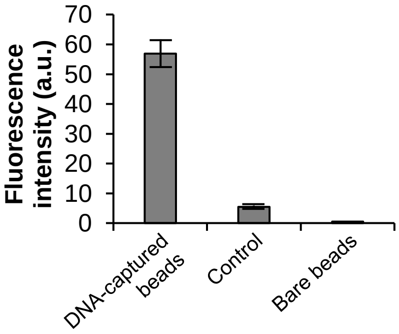

Fig. S6. Removal of residual ssDNA strands from the amplification chamber. An amplification chamber previously used for PCR was washed thoroughly with 0.2 M NaOH and buffer, and then filled with reverse primer-coated beads while the selection process was carried out in the selection chamber. Target binding ssDNA strands were electrophoretically transferred back to the amplification chamber where PCR occurred. As a control, bead-based PCR was performed on a washed chamber without transferring target-binding ssDNA to the amplification chamber. The fluorescence intensity of the beads when DNA is transferred to the amplification is significantly stronger than the beads in the control experiment suggesting successful removal of residual DNA from the amplification chamber following washing.

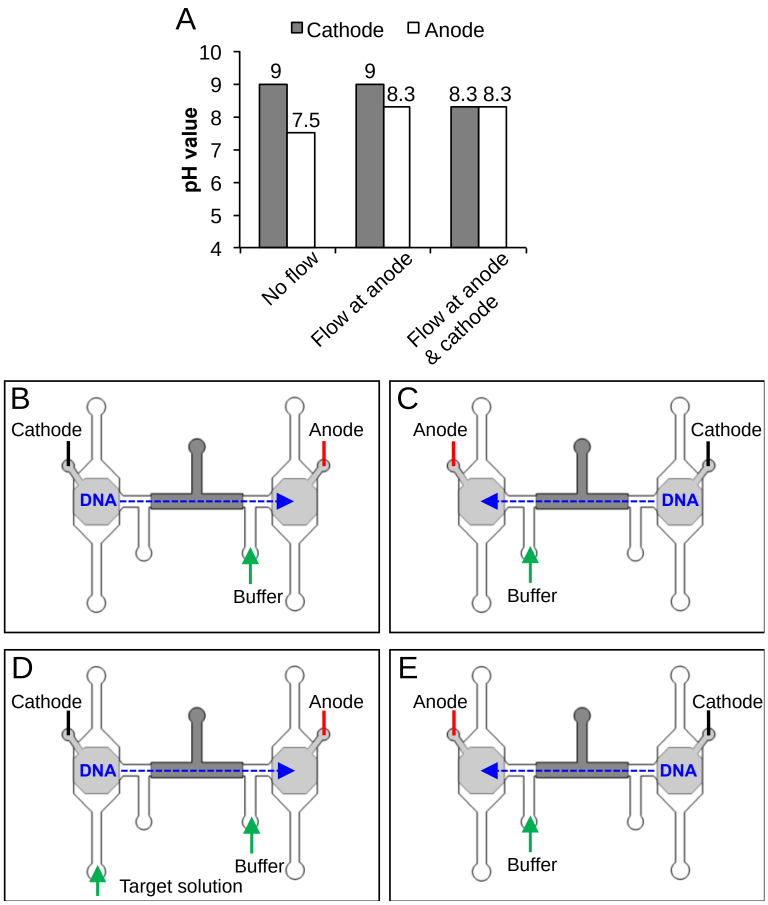

Fig. S7. (*A*) Changes in pH levels in the chambers of the microchip inserted with the anode and cathode during electrophoretic transfer of DNA. Schematics of pH control in the chips during electrophoretic transfer of ssDNA strands. Buffer is introduced into the chips (flow rate: 1 μL/min) to minimize the pH changes in the chambers. In the experiment for IgE protein target, buffer is introduced into (*B*) the amplification chamber and (*C*) the selection chamber through the supplementary inlets during transfer of DNA strands to the direction indicated by the dotted lines. In the experiment for the bisboronic

acid sensor-glucose mixture, (*D*) buffer is introduced into the amplification chamber while solution containing target molecules is injected into the selection chamber during DNA transfer into the amplification chamber. (*E*) Buffer is introduced into the selection chamber through an inlet during DNA transfer into that chamber.

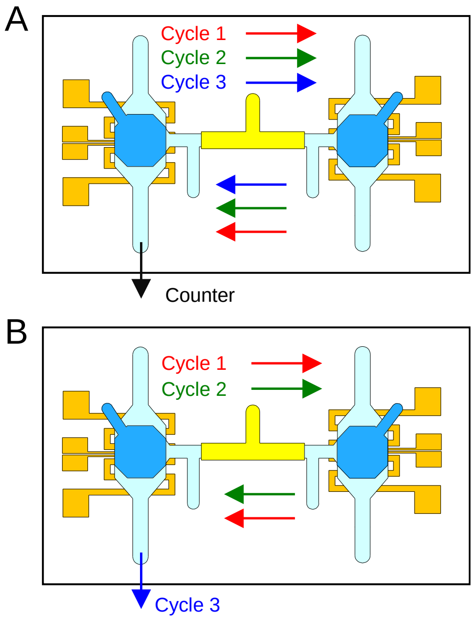

Fig. S8. Schematics showing the multiple SELEX cycles continuously performed using our chips. (*A*) Using human IgE protein as target, 3 SELEX cycles were continuously carried out followed by 1 additional counter selection process before collecting the final enriched DNA sample. (*B*) For enrichment of the bisboronic acid sensor-glucose mixture binding strands, 3 cycles of continuous SELEX were performed.

Table S1. Microfludic SELEX procedure for IgE.

Step No.	Step Description	Flow Rate (µL/min)	Volume (µL)	Oligonucleotide Location
1	Introduce PBS buffer into both chambers			
2	Introduce selection beads into the selection chamber until approximately 50% of the chamber is filled with beads			
3	Introduce amplification beads into the amplification chamber until approximately 50% of the chamber is filled with beads			
4	Introduce buffer containing randomized library into the selection chamber	10	100	Selection chamber
5	Introduce buffer into the selection chamber to remove weakly binding oligonucleotides	20	330	Selection chamber
6	Increase selection chamber temperature to 57 C and apply 25 V/cm electric field with positive terminal in the amplification chamber for 25 minutes while infusing (1 µL/min) fresh selection buffer into the amplification chamber.			Transitioning to Amplification chamber
7	Introduce PCR reagents			Amplification chamber
8	Perform 25 cycles of PCR thermal cycling			Amplification chamber
9	Introduce selection buffer containing 0.2 M NaOH into the amplification chamber			Amplification chamber
10	Remove and introduce fresh selection beads until approximately 50% of the chamber is filled with beads			Amplification chamber
11	Apply 25 V/cm electric field with positive electrode in the selection chamber for 25 minutes while infusing (1 µL/min) fresh selection buffer into the selection chamber.			Transitioning to selection chamber
12	Remove and introduce fresh amplification beads into the amplification chamber until approximately 50% of the chamber is filled with beads			Selection chamber
13	Repeat steps 5 to 12 1 time			
14	Introduce buffer to remove weakly binding oligonucleotides	20	330	Selection chamber
15	Increase selection chamber temperature to 57 C and apply 25 V/cm electric field with positive terminal in the amplification chamber for			Transitioning to amplification chamber
16	Introduce PCR reagents			Amplification chamber
17	Perform 25 cycles of PCR thermal cycling			Amplification chamber
18	Introduce selection beads with counter target (IgG) into the selection chamber			Amplification chamber
19	Introduce selection buffer containing 0.2 M NaOH into the amplification chamber			Amplification chamber
20	Apply 25 V/cm electric field with positive electrode in the selection chamber for 25 minutes			Transitioning to Selection chamber
21	Introduce buffer into the selection chamber to wash beads and collect buffer exiting the device which contains eluted oligonucleotides	20	330	Selection chamber

Table S2. Microfludic SELEX procedure for glucose-boronic acid mixture.

Step No.	Step Description	Flow Rate (µL/min)	Volume (µL)	Oligonucleotide Location
1	Introduce PBS buffer into both chambers			
2	Introduce capture beads into the selection chamber until approximately 50% of the chamber is filled with beads			
3	Introduce amplification beads into the amplification chamber until approximately 50% of the chamber is filled with beads			
4	Introduce buffer containing randomized library into the selection chamber			
5	Introduce buffer into the selection chamber to remove oligonucleotides not captured by the anchor beads	20	66	
6	Introduce buffer containing a counter target (bis-boronic acid)	20	330	
7	Introduce buffer containing target molecule to elute remaining oligonucleotides anchored to bead surfaces	10	100	Selection chamber
8	Apply 25 V/cm electric field with positive terminal in the amplification chamber for 25 minutes while infusing (1 µL/min) fresh selection buffer into the amplification chamber.			Transitioning to amplification chamber
9	Introduce PCR reagents			Amplification chamber
10	Perform 25 cycles of PCR thermal cycling			Amplification chamber
11	Introduce selection buffer containing 0.2 M NaOH into the amplification chamber			Amplification chamber
12	Remove and introduce fresh capture beads until approximately 50% of the chamber is filled with beads			Amplification chamber
13	Apply 25 V/cm electric field with positive electrode in the selection chamber for 25 minutes while infusing (1 µL/min) fresh selection buffer into the selection chamber.			Transitioning to selection chamber
14	Remove and introduce fresh amplification beads into the amplification chamber until approximately 50% of the chamber is filled with beads			Selection chamber
15	Repeat steps 5 to 15 2 times			
16	Introduce buffer into the selection chamber to remove oligonucleotides not captured by the anchor beads	20	66	Selection chamber
17	Introduce buffer containing a counter target (bis-boronic acid)	20	330	Selection chamber
18	Introduce buffer containing target molecule to elute remaining oligonucleotides anchored to bead surfaces while collecting the eluted oligonucleotides exiting the device	10	100	Selection chamber

		Sequences of selected target-binding strands (5’ → 3’)
IgE protein	SIGE1	TGTGCATACTGACAATTGCTTTGCTGTTCCTCATTACGTG
	SIGE2	GTCCAGTATCTGCCATTCTTGTCACTAAGCCTCGTGTTAG
	SIGE3	CGTGGACGTAGACCCGTATGCCGAAATAAGCTTATGTGCT
	SIGE4	TCAGTACGCGTTTGACTCGATCCGAGGTCTCCCTTTGGTG
	SIGE5	GATGTAGGCCATCCTGTGGGGGTACGGGGCGGGCTGTGGT
	SIGE6	GCACGTTTATCCGTCCCTCCTAGTGGCGTGC
	SIGE7	ACTAGACCGGGAGCAAAACGGCAGATTGGGGCAAGGCCGG
	SIGE8	GCACGTTTATCCGTCCCTCCTAGTGGCGTGC
	SIGE10	GGGCACCTGGGCAGGCCGAAAGTGACACGGGGTGTGGACC
	SIGE11	AAGTGCCCATGCTATGCTACTATGAGAAGTAGTTCGGACC
	SIGE12	TACTCCACACACCGTAGGGTGACTTTGGTGCCCCGTGGCC
	SIGE13	TTGGGTGGATTATAACATTATATCCGTCTTTTTCGACTAG
	SIGE14	GTTCGGAGTACTCAAGATTCTTTTACTTAACGTTAACACC
	SIGE15	TACAATGTTTCATAGCTCGGCGTCGATTCGTGGCTTTTGG
	SIGE16	ACACGTACCGCAGCTACAATAAGTTAATGGGTGCGCGCCC
	SIGE17	GATACATTGAAGTAGGGGAGCGAGTCTGTTCGCGGACGGG
	SIGE18	TGCAGTACAGTTCTTTTGTTGTAGACAGGGTTGACACTGG
Bisboronic acid sensor-glucose mixture	SGB 1	TCGGGACGACGGAACGGAGAAAAATCTCATCGTGTCGGCAGTCGTCCCGA
	SGB 2	TCGGGACGACCAACCAGAGAAAAATCCCAGTTTCTAGGTAGTCGTCCCGA
	SGB 3	TCGGGACGACCAACCAGAGAAAAATCCCAGTTTCTAGGTAGTCGTCCCGA
	SGB 4	TCGGGACGACCCATCGCATTTCACACTAAATCCCGGCAAAGTCGTCCCGA
	SGB 5	TCGGGACGACGGGTCCGTTAGTAACCTTTCTTATGGGGCGGTCGTCCCGA
	SGB 6	TCGGGACGACGCGTAACGAAGATTTATTTCGTTCGTATCTGTCGTCCCGA
	SGB 7	TCGGGACGACGGCACCCGACGGCGGAAGGTGTAAGTGACGGTCGTCCCGA

Figure S9. Sequences of randomly selected oligonucleotides collected from the continuous SELEX experiments. Primer regions are excluded from the sequences. Sequences 1-18 and sequences 19-25 were identified following experiments with IgE protein and the bisboronic acid sensor-glucose mixture, respectively.

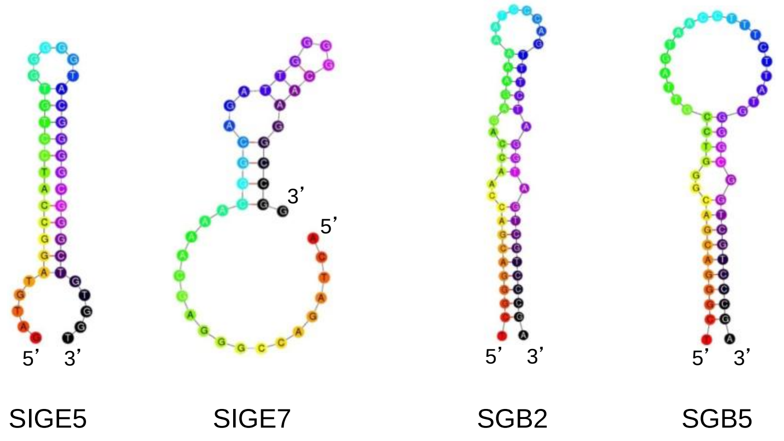

Fig. S10. Secondary structures of strands selected for binding affinity studies. The DNA folding freeware was used to predict the structures of the sequences [S1]. SIGE5 and

SIGE7 are oligonucleotides isolated against IgE. SGB2 and SGB5 are oligonucleotides isolated against molecules in the bisboronic acid sensor-glucose mixture.

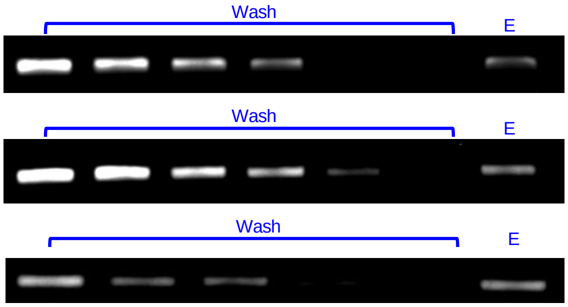

Fig. S11. Gel electropherograms of eluates from affinity selection against IgE. Oligonucleotides removed from successive washes shown in the lane Wash. Binding oligonucleotides shown in lane E.

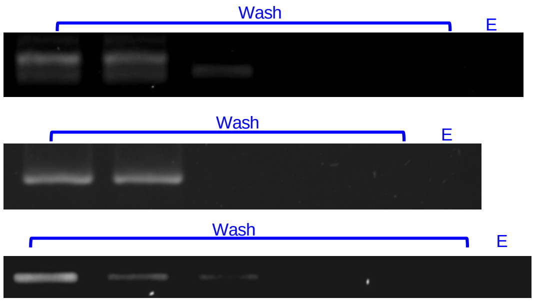

Fig. S12. Gel electropherograms of collected eluates from affinity selection against IgG (as control for IgE), showing no significant binding. Oligonucleotides removed from successive washes shown in Wash lanes. Binding oligonucleotides shown in lane E.

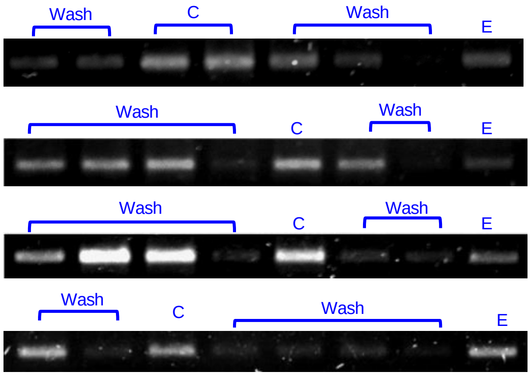

Fig. S13. Gel electropherograms of collected eluates from affinity selection against glucose-boronic acid mixture. Lane Wash: successive washes; Lane E: elution; Lane C: counter selection.

Supplementary References

[S1] M. Zuker, "Mfold web server for nucleic acid folding and hybridization prediction," *Nucleic Acids Research*, vol. 31, pp. 3406-3415, 2003.
